# Investigating the metabolic reprogramming mechanisms in diabetic nephropathy: a comprehensive analysis using bioinformatics and machine learning

**DOI:** 10.3389/fcell.2025.1630708

**Published:** 2025-08-29

**Authors:** Shan He, Yi Wei Chen, Jian Ye, Yu Wang, Qin Kai Chen, Si Yi Liu

**Affiliations:** ^1^ Department of Nephrology, The First Affiliated Hospital, Jiangxi Medical College, Nanchang University, Nanchang, China; ^2^ Department of Orthopaedics, Jiujiang University Affiliated Hospital, Jiujiang, China

**Keywords:** GEO database, diabetic nephropathy, metabolic reprogramming, bioinformatics, qRT-PCR

## Abstract

**Background:**

Diabetic nephropathy (DN) is a common complication of diabetes, characterized by damage to renal tubules and glomeruli, leading to progressive renal dysfunction. The aim of our study is to explore the key role of metabolic reprogramming (MR) in the pathogenesis of DN.

**Methods:**

In our study, three transcriptome datasets (GSE30528, GSE30529, and GSE96804) were sourced from the Gene Expression Omnibus (GEO) database. These datasets were integrated for batch effect correction and subsequently subjected to differential expression analysis to identify differentially expressed genes (DEGs) between DN and control samples. The identified DEGs were cross-referenced with genes associated with MR to derive MR associated differentially expressed genes (MRRDEGs). These MRRDEGs underwent Gene Ontology (GO) and Kyoto Encyclopedia of Genes and Genomes (KEGG) enrichment analyses. To identify key genes and develop diagnostic models, four machine learning algorithms were employed in conjunction with weighted gene co-expression network analysis (WGCNA) and the protein interaction tool CytoHubba. Gene set enrichment analysis (GSEA) and CIBERSORT analysis were conducted on the key genes to assess immune cell infiltration in DN. Additionally, a competitive endogenous RNA (ceRNA) network was constructed using the key genes. Finally, the expression levels of core genes in human samples were validated through quantitative real-time PCR (qRT-PCR).

**Results:**

We identified 256 MRRDEGs, highlighting metabolic and inflammatory pathways in DN. KEGG analysis linked these genes to the MAPK signaling pathway, suggesting its key role in DN. Six key genes were pinpointed using WGCNA, PPI, and machine learning, with their diagnostic value confirmed by ROC analysis. CIBERSORT revealed a strong link between these genes and immune cell infiltration, indicating the immune response’s role in DN. GSEA showed these genes’ involvement in inflammatory and metabolic processes. A ceRNA network was predicted to clarify gene regulation. qRT-PCR confirmed the expression patterns of *CXCR2, NAMPT*, and *CUEDC2*, aligning with bioinformatics results.

**Conclusion:**

Through bioinformatics analysis, a total of six potential MRRDEGs were identified, among which *CUEDC2*, *NAMPT*, *CXCR2* could serve as potential biomarkers.

## 1 Introduction

Diabetic nephropathy (DN) is a severe complication associated with diabetes mellitus (Thipsawat, 2021), characterized by progressive damage to the renal tubules and glomeruli, ultimately leading to a decline in kidney function (Levin et al., 2020). It is estimated that approximately 30%–40% of patients with diabetes will develop DN (Thomas et al., 2015), making it a significant contributor to end-stage renal disease (ESRD) and associated mortality (Gilbertson et al., 2005; Cross et al., 2021; Dw et al., 2025). Despite the implementation of blood glucose regulation and therapeutic measures aimed at diminishing urinary albumin excretion, the fundamental progression of DN remains largely unaltered (Thomas et al., 2015). Recently, researchers have been exploring new treatments like glucose stabilizers, kidney-protective agents, and therapies targeting inflammation and fibrosis (Ghose et al., 2024). SGLT2 inhibitors, for instance, help regulate blood glucose and may reduce kidney workload by decreasing glucose reabsorption (Neuen et al., 2024). Despite these advances, current treatments have limitations in slowing disease progression and cannot fully reverse DN. Understanding the molecular mechanisms behind DN could help identify new therapeutic targets and improve prevention and management strategies.

The pathogenesis of DN is intricate, with recent research highlighting the pivotal role of metabolic reprogramming (MR) in various diseases, such as tumors and metabolic disorders (Li and Liao, 2021; Huang et al., 2023). Metabolic reprogramming refers to the cellular adaptation to environmental changes through the modification of metabolic pathways under specific pathological conditions (Li et al., 2021). Recent studies have underscored the significance of MR in DN, demonstrating its close association with the onset and progression of the disease. Notably, significant alterations have been observed in energy and lipid metabolism (Wang et al., 2022a; Yu et al., 2025). A fundamental component of metabolic reprogramming in DN is the modification of mitochondrial function. Mitochondria are central to cellular energy metabolism, and their impaired function leads to increased oxidative stress, apoptosis, and dysregulated autophagy, thereby facilitating the advancement of renal fibrosis and dysfunction (Fan et al., 2024; Sharma et al., 2013). This dysfunction not only accelerates the progression of DN but also involves the reprogramming of lipid metabolism. Under diabetic conditions, renal cells exhibit significant lipid metabolic abnormalities, including increased lipid uptake, impaired fatty acid oxidation, disrupted cholesterol efflux, and enhanced lipid catabolism (Yu et al., 2025). These alterations result in the accumulation of lipids such as free fatty acids, diacylglycerol, and ceramides, which subsequently induce lipotoxicity, inflammation, and fibrosis (Yu et al., 2025). Additionally, the interplay between MR and DN is critical, as metabolic dysregulation not only exacerbates renal injury but also promotes the progression of the disease through mechanisms such as oxidative stress and inflammation (Hou et al., 2025).

Existing research has highlighted the critical role of MR in the pathological progression of DN. However, the regulatory mechanisms at the molecular level and the specific role of MR in DN remain insufficiently understood. Consequently, this study aims to elucidate the key characteristics and functional importance of MR in DN through bioinformatics analysis. We employed bioinformatics and machine learning methodologies to identify biomarkers associated with metabolic reprogramming in DN. Initially, we acquired the DN dataset from the GEO database and conducted differential gene expression analysis. This was followed by an intersection with genes related to metabolic reprogramming. We further integrated weighted gene co-expression network analysis (WGCNA), four distinct machine learning algorithms, and protein-protein interaction (PPI) network construction to pinpoint key differentially expressed genes associated with MR. Subsequently, a diagnostic model for DN was developed, and the diagnostic efficacy of the model and key genes was validated. Additionally, immune cell infiltration analysis was conducted to investigate the relationship between key genes and immune cell populations. The pivotal genes were employed to construct competitive endogenous RNA (ceRNA) networks. Finally, the expression levels of these key genes were experimentally validated. These findings have the potential to offer novel insights into the early diagnosis and therapeutic strategies for DN.

## 2 Materials and methods

### 2.1 Data acquisition

We procured gene expression data pertinent to diabetic nephropathy from the GEO database (Barrett et al., 2013), specifically datasets GSE30528 (Woroniecka et al., 2011), GSE30529 (Woroniecka et al., 2011), and GSE96804 (Pan et al., 2018; Shi et al., 2018), utilizing the GEO query package. The GSE30528 and GSE30529 dataset is derived from the GPL571 platform, while the GSE96804 dataset is based on the GPL17586 platform. The GSE30528 dataset comprises 13 control samples and 9 DN samples, whereas the GSE96804 dataset includes 20 healthy human renal tubular samples and 41 tubular samples from DN patients. Additionally, the GSE30529 dataset consists of 10 control samples and 12 DN samples. We employed the R package ‘sva’ (v3.50.0) to adjust for batch effects across the diabetic nephropathy datasets GSE30528, GSE30529, and GSE96804, resulting in a consolidated dataset, DN_Datasets, which encompasses 62 DN samples and 43 control samples. The dataset specifics are outlined in Table 1. Furthermore, we identified 1,468 Metabolic Reprogramming-Related Genes (MRRGs) from the GeneCards database (Stelzer et al., 2016) (http://www.genecards.org) by searching for protein-coding genes related to “metabolic reprogramming” with a relevance score >4.

**TABLE 1:** GEO Dataset Information list.

Accession	GSE30528	GSE96804	GSE30529
Platform	GPL571	GPL17586	GPL571
Experiment type	Expression profiling by array	Expression profiling by array	Expression profiling by array
Species	*Homo sapiens*	*Homo sapiens*	*Homo sapiens*
Tissue	glomeruli	glomeruli	glomeruli
Samples in Control group	Control (13)	Control (20)	Control (10)
Samples in Disease group	DN (9)	DN (41)	DN (12)
Reference	Transcriptome analysis of human diabetic kidney disease	Dissection of Glomerular Transcriptional Profile in Patients With Diabetic Nephropathy: SRGAP2a Protects Podocyte Structure and Function	Transcriptome analysis of human diabetic kidney disease

DN, diabetic nephropathy.

### 2.2 Differentially expressed gene analysis

To identify the differentially expressed genes (DEGs) associated with DN, we employed the ‘limma’ (v3.58.1) package to conduct a differential analysis of the expression profile data from the DN_Datasets. The criteria for selecting DEGs were set at |logFC| > 0.25 and p. value <0.01. To further identify metabolic reprogramming-related differentially expressed genes (MRRDEGs), we intersected the DEGs derived from the DN_Datasets analysis with MRRGs. This intersection was visualized using a Venn diagram, and the resultant MRRDEGs were used for subsequent analyses. Additionally, a volcano plot was generated using the R package ggplot2 (v3.5.1) to illustrate the differential analysis results. For functional annotation, Gene Ontology (Mi et al., 2019) (GO) and Kyoto Encyclopedia of Genes and Genomes (Kanehisa and Goto, 2000) (KEGG) pathway analyses were conducted using the ‘clusterProfiler’ (v4.10.1) package (Yu et al., 2012), with an enrichment significance threshold set at p. adjust <0.05, corrected using the Benjamini–Hochberg (BH) method.

### 2.3 Weighted gene Co-Expression network analysis (WGCNA)

WGCNA is a computational algorithm designed to cluster genes into distinct modules and elucidate the relationships between these modules and disease characteristics. To thoroughly investigate the genetic mechanisms underlying the pathogenesis of DN, a co-expression network was constructed utilizing the ‘WGCNA’ (v1.72-5) package (Langfelder and Horvath, 2008) in the R. This network was developed using the top 40% of genes with the highest variance from the DN_Datasets dataset. A dynamic tree cut method was employed to merge modules, applying a threshold of 0.15. Additional criteria for constructing the co-expression network included the use of the ‘pickSoftThreshold’ function, which selects powers of soft thresholds (β) based on a scale-free topology criterion (independence index R^2^ = 0.8) (Tanimura et al., 2006), and a minimum module size of 100 genes. Spearman correlation analysis was conducted to identify potential associations between the modules and DN. The intersection of MRRDEGs and modular genes was obtained by venn diagram to identify hub MRRDEGs.

### 2.4 Construction of protein-protein interaction (PPI) networks

Protein-protein interaction (PPI) networks were developed utilizing the STRING online database (Szklarczyk et al., 2019) (https://www.string-db.org/), with a confidence interaction score threshold set at 0.4 to establish significance. The construction and analysis of the PPI network were conducted using Cytoscape (v3.9.1) software (Shannon et al., 2003). Within Cytoscape, the CytoHubba (C et al., 2014) plugin was employed to identify key genes. In particular, the Maximal Clique Centrality (MCC) algorithm was successful in identifying core genes with high centrality, whereas the Degree method enabled the identification of genes with the most connections. The Protein-Protein Interaction (PPI) network was analyzed using MRRDEGs scores, and the top 80 MRRDEGs were selected based on these scores. This approach focuses on genes with the highest scores, facilitating manageable downstream analysis and highlighting key network components. A venn diagram was employed to illustrate the overlap of genes identified by the two algorithms, thereby pinpointing the candidate genes.

### 2.5 Machine learning

For the identification of key genes in the diagnosis of DN, we utilized four machine learning algorithms: Random Forest (RF) (Rigatti, 2017), Extreme Gradient Boosting (XGB) (Guo and Chang, 2022), Support Vector Machine (SVM) (Tan et al., 2014), and Generalized Linear Model (GLM) (Song et al., 2013). RF was selected for its robust capability in feature importance ranking, XGB for its proficiency in capturing non-linear interactions pertinent to metabolic reprogramming, SVM with a radial basis function (RBF) kernel for its effectiveness in high-dimensional classification tasks, and GLM as an interpretable baseline model. Using the train function from the R ‘caret’ (v6.0-94) package, we trained RF, SVM, XGB, and GLM models, utilizing the ‘randomForest' (v4.7-1.1), ‘kernlab' (v0.9-33), ‘xgboost' (v1.7.8.1), and ‘stats' (v4.3.3) packages, respectively. Concurrently, we utilized the ‘caret’ (v6.0-94) package’s to tune their parameters through grid search, and evaluated their performance using fivefold cross-validation. Furthermore, to ensure the reliability of the models, we generated residual boxplots, feature importance plots, reverse cumulative distribution of residuals, and receiver operating characteristic (ROC) curves for the models.

### 2.6 Diagnostic model construction and assessment

To determine the feasibility of this diagnostic model as a diagnostic factor, a nomogram model was performed for six genes using the ‘rms’ (v6.7-1) package. The reliability of the model predictions was then assessed using ROC curves. To evaluate the predictive performance and clinical applicability of the models, calibration curves and decision curve analysis (DCA) were employed. Furthermore, based on the results of the DCA, we assessed the clinical impact curves (CIC).

### 2.7 Immune infiltration analysis

The immune infiltration matrix was derived from the DN gene expression dataset using the ‘CIBERSORT’ (v0.1.0) package in R. The CIBERSORT algorithm was then used to compare the distribution of immune cell infiltration between patients with DN and controls (Newman et al., 2015). Additionally, Spearman’s correlation analysis was conducted to explore the relationship between MRRDEGs, DN, and immune infiltration. The immune infiltration matrix for each sample and group was visualized using the ‘ggplot2’ (v3.5.1) package.

### 2.8 Single-sample gene set enrichment analysis (ssGSEA)

We employed single-sample Gene Set Enrichment Analysis (ssGSEA), an advanced analytical technique that leverages molecular feature databases, to elucidate the effects of gene expression (Subramanian et al., 2005). The calculations were conducted using the ‘gsva’ function within the clusterProfiler package (v4.10.1). This method utilizes a precise algorithm for functional enrichment analysis, allowing us to investigate the potential biological pathways associated with key MRRDEGs.

### 2.9 ceRNA networks and validation of key gene expression

To predict microRNAs (miRNAs) targeting the model genes, we utilized TargetScan (http://www.targetscan.org/) with a screening criterion of pancancer num >7. For predicting lncRNA-miRNA interactions, we employed Starbase (https://starbase.sysu.edu.cn/starbase2/) using a screening criterion of pancancer num >12. Following this, we utilized Cytoscape (v3.9.1) software to construct and visualize the lncRNA-miRNA-mRNA regulatory networks. To assess the differential expression of crucial genes between disease and control groups within the DN_Datasets, we employed the Mann-Whitney U test (Wilcoxon rank-sum test). The results of this differential analysis were visualized using group comparison graphs generated with the R package ggplot2 (v3.5.1).

### 2.10 qRT-PCR experiments

Additionally, qRT-PCR validation was conducted. Between March to April 2025, twelve whole-blood samples were collected from the First Affiliated Hospital of Nanchang University, including six from DN patients and three from healthy controls, aged 30 to 60. All participants gave informed consent, and the study was approved by the ethics committee (Ethical number: (2024) CDYFYYLK (07–026)). Blood samples ranging from 3 to 5 mL were collected into EDTA tubes for subsequent white blood cell enrichment. Total leukocytes were isolated through the lysis of red blood cells using ACK Lysing Buffer (Thermo Fisher, USA). The whole blood was combined with 10 volumes of the lysis buffer, incubated at room temperature for 10 min, and then subjected to centrifugation at 300 *g* for 5 min. The resulting leukocyte pellet underwent three washes with PBS to ensure the thorough removal of erythrocyte debris. RNA was extracted from both DN patients’ and healthy controls’ samples, with DN samples taken before treatment. Reverse transcription was performed using the Servicebio® RT First Strand cDNA Synthesis Kit from Wuhan, China, followed by quantitative PCR. In the next step, qPCR was performed using the 2 × SYBR Green qPCR Master Mix (None ROX) from Servicebio, Wuhan, China, following the manufacturer’s instructions. The thermocycling protocol included an initial phase at 95 °C for 5 min, followed by 40 cycles of 95 °C for 10 s and 60 °C for 30 s β-actin was used as the reference gene for data normalization, and gene expression was calculated using the 2^−ΔΔCT^ method. The primers used are listed in Table 2.

**TABLE 2:** Primer sequences for quantitative real-time PCR.

Gene Names	Forward (5′-3′)	Reverse (3ʹ-5ʹ)
CXRC2	CTCCCTTTCATAGGTCACAG	AAACTTAAATCCTGACTGGGTC
CUEDC2	GGAACAAAGAGAACCTGCA	CTTCTTTGAGCATTTCGGG
NAMPT	GAAATGTTCTCTTCACGGTGG	GACTGAACAAGAATAGTCTCAATCC
ATF3	AGAAGGAGAAGACGGAGTG	TATGCAGGTCTTCAGGACC
GDF15	GCTGGGAAGATTCGAACAC	ACTTCTGGCGTGAGTATCC
CEBPD	AGAAGTTGGTGGAGCTGTC	GCAGCTGCTTGAAGAACTG

### 2.11 Statistical analysis

All statistical analyses were performed using R software, version 4.3.3. For comparing two groups with normally distributed data, we applied the Student’s t-test. The chi-square test was used to compare categorical and pairwise features across different groups. The Mann-Whitney U test was employed to determine statistically significant differences between two groups, while the Kruskal–Wallis test was used to assess significant differences among multiple independent groups. Pearson’s correlation test was applied to evaluate correlations between normally distributed variables, and Spearman’s correlation test was used for non-normally distributed variables. All statistical tests were two-sided, and a p-value of less than 0.05 was considered statistically significant unless otherwise specified.

## 3 Results

### 3.1 Calibration of data set, variance analysis, and functional enrichment analysis

**FIGURE 1:**
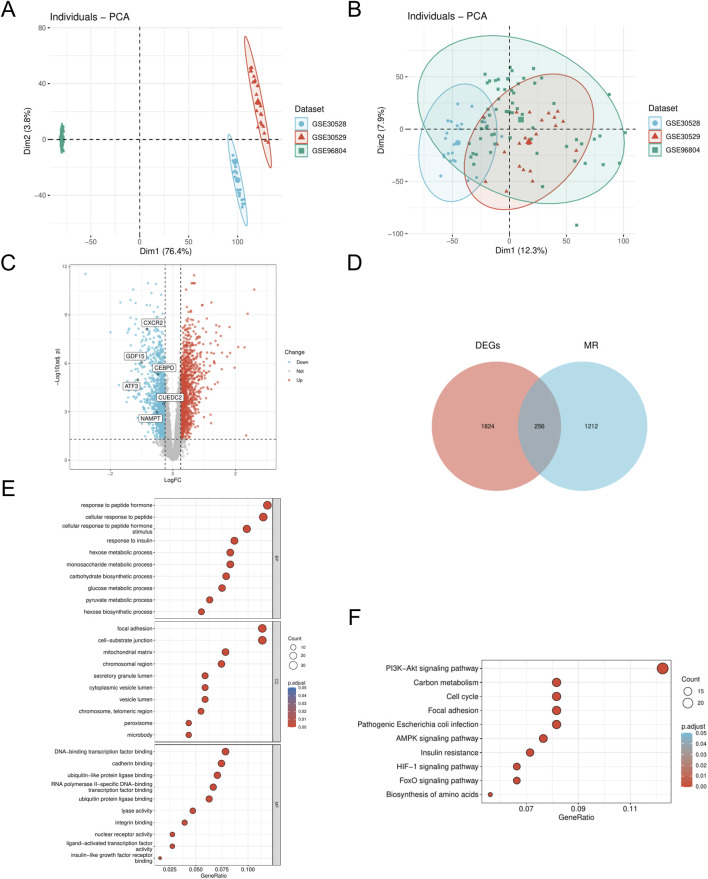
Dataset correction and analysis of differentially expressed genes **(A)**. PCA plot of DN- Datasets before correction. **(B)** PCA plot of the corrected DN-Datasets. **(C)** Volcano plot of differential analysis results between DN and Control groups in the DN- Datasets dataset. **(D)** Venn diagram of the DEGs and MR. **(E)** GO enrichment analyses of MRRDEGs. **(F)** KEGG enrichment analyses of MRRDEGs. DN, Diabetic nephropathy. MR, Metabolic reprogramming. GO, Gene ontology. KEGG, Kyoto encyclopedia of genes and genomes. BP, Biological process. CC, Cellular component. MF, Molecular function. The screening criteria for GO/KEGG enrichment items were p. Adj <0.05 and FDR value (q. value) < 0.25, and the p value correction method was Benjamini–Hochberg (BH).

We employed the R package ‘sva' (v3.50.0) to address batch effects in the DN_Datasets by performing de-batching, resulting in a refined dataset. We then conducted a comparative analysis of the datasets before and after batch effect removal using Principal Component Analysis (PCA) plots (Figures 1A,B). The PCA results indicate that the batch effect in the DN_Datasets was effectively mitigated. To identify differentially expressed genes (DEGs) associated with DN across various subgroups, we utilized the R package ‘limma' (v3.58.1) to analyze the expression profiling data of the DN_Datasets. This analysis revealed a total of 2,080 DEGs, comprising 1,007 upregulated and 1,073 downregulated genes in the DN group compared to the control group. Subsequently, a volcano plot (Figure 1C) was generated to visualize the differential analysis results. To identify the MRRDEGs, we determined the intersection of DEGs from the DN_Datasets, which pertains to DN, with MRRGs. This process yielded a total of 256 genes designated as MRRDEGs for further analysis. A venn diagram was constructed to illustrate these results (Figure 1D). GO functional analysis revealed that the biological processes (BPs) were predominantly enriched in pathways such as response to insulin, hexose metabolic process, monosaccharide metabolic process, carbohydrate biosynthetic process, and glucose metabolic process. The cellular components (CC) were primarily associated with the mitochondrial matrix and chromosomal region. Regarding molecular function (MF), significant enrichment was observed in DNA-binding transcription factor binding, calmodulin binding, ubiquitin-like protein ligase binding, and transcription factor binding (Figure 1E). The KEGG pathway analysis indicated significant enrichment in the AMPK signaling pathway, insulin resistance, cell cycle, and PI3K-Akt signaling pathway (Figure 1F).

### 3.2 Weighted gene Co-expression network analysis (WGCNA)

**FIGURE 2:**
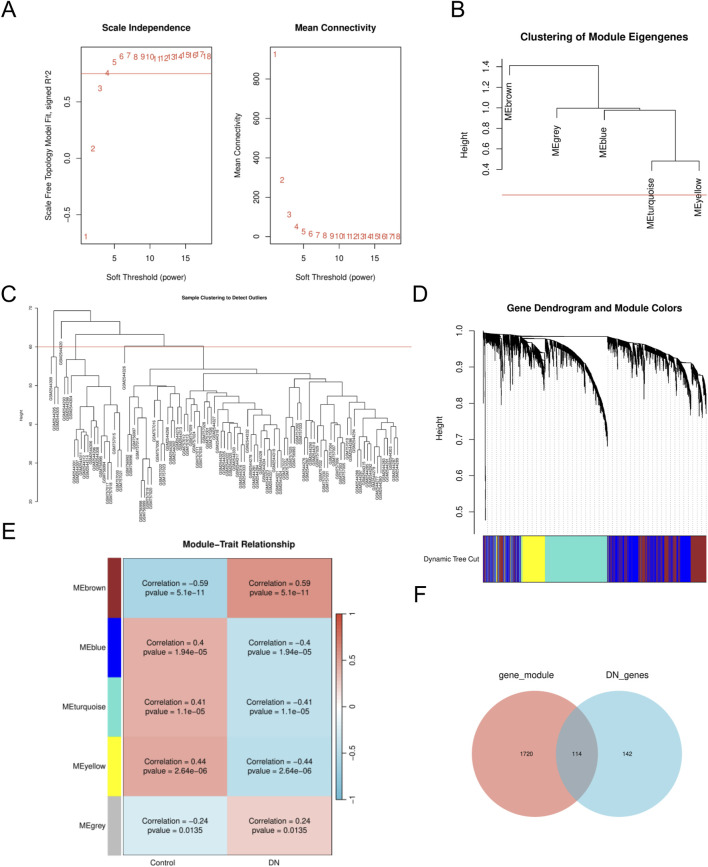
The WGCNA analysis and identification of MRRDEGs **(A)**. The soft threshold power and the mean connectivity of WGCNA. **(B)** Clustering dendrogram of genes. **(C)** A cluster tree. **(D)** Gene dendrograms from average linkage hierarchical clustering. **(E)** Module-trait relationships. **(F)** Venn diagram of the MRRDEGs and module genes. MRRDEGs, Metabolic reprogramming related differentially expressed genes. WGCNA, Weighted Gene Co-expression Network Analysis.

To identify key gene modules associated with DN, we employed the WGCNA algorithm to construct co-expression networks and modules for both DN and control groups. Initially, we calculated the expression variance of each gene within the DN datasets and selected the top 40% of genes exhibiting the highest variance for further analysis. A threshold of β = 10 (scale-free R = 0.8) was utilized to construct a scale-free network, facilitating the identification of co-expression gene modules (Figure 2A). Through hierarchical clustering of the samples, eight distinct co-expression modules, each represented by a unique color, were identified using the dynamic tree cut algorithm (Figures 2B–D). Subsequently, we assessed the co-expression similarity and adjacency of these modules in relation to clinical characteristics of the control and DN groups. Our analysis revealed that the blue and yellow modules were strongly associated with DN, encompassing a total of 1,834 genes (Figure 2E). Additionally, we examined the extent of overlap between module genes and MRRDEGs, identifying 114 genes in common (Figure 2F).

### 3.3 Protein-protein interaction (PPI) networks

**FIGURE 3:**
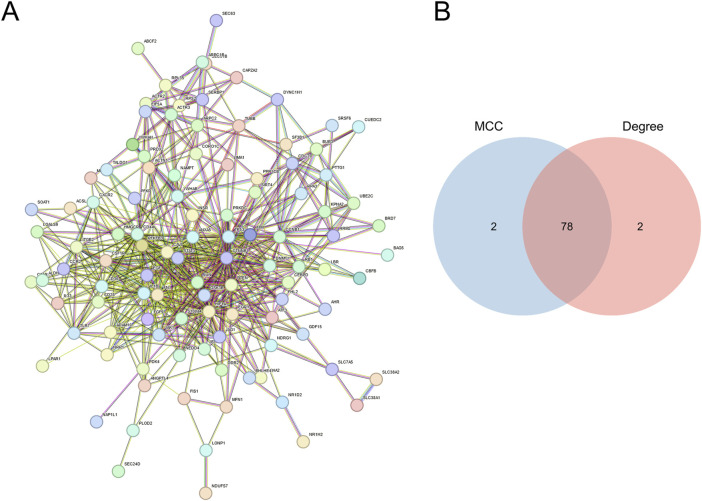
Identification of mitochondria-related DEGs **(A)**. PPI networks of overlapping MRRDEGs constructed in the STRING database. **(B)** Venn diagram of hub MRRDEGs identified by two different algorithms in Cytohubba (MCC and Degree).

PPI networks (Figure 3A) were developed utilizing the STRING database (https://www.string-db.org/) for the 118 genes obtained in the previous step. The construction and analysis of the PPI network were conducted using Cytoscape software. Within Cytoscape (v3.9.1), the CytoHubba plugin was employed to apply two distinct computational methods, namely, Maximal Clique Centrality (MCC) and Degree, for the identification of key genes. The scores are detailed in Supplementary Table S1, S2. Subsequently, a total of 78 key genes (Supplementary Table S3), referred to as hub MRRDEGs, were identified through the use of a Venn diagram (Figure 3B).

### 3.4 Machine learning

**FIGURE 4:**
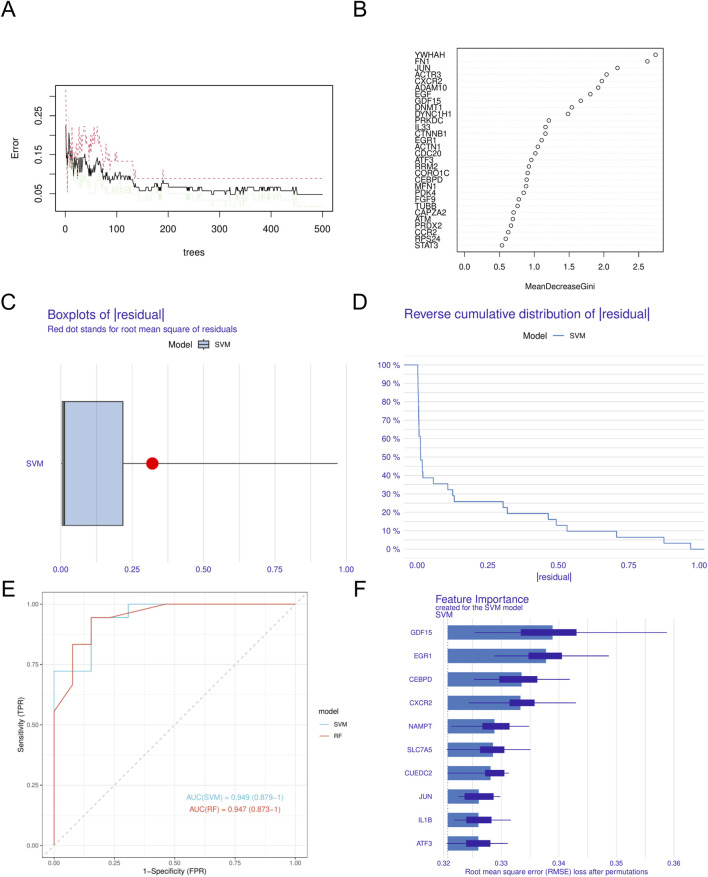
Construction of machine learning models **(A)**. Confidence intervals for error rates of random forest models **(B)**. The relative importance of genes in random forest models. **(C)** Boxplots showed the residuals of SVM model. **(D)** Cumulative residual distribution of SVM model. **(E)** ROC analysis of SVM and RF models based on five-fold cross-validation in the testing cohort. **(F)** The important features in SVM models. DN, Diabetic nephropathy. SVM, Support Vector Machine.

**FIGURE 5:**
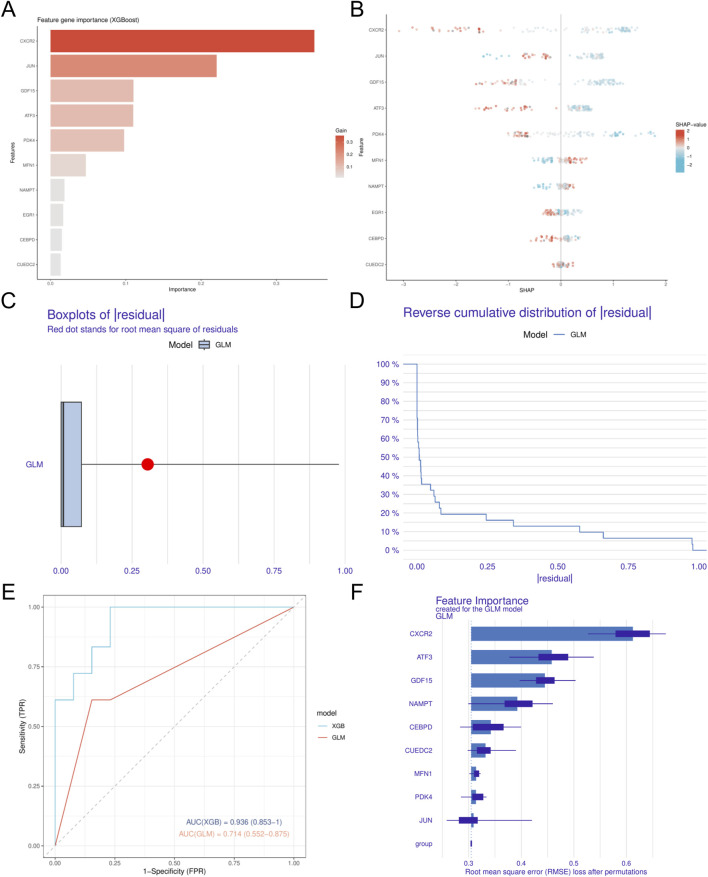
Identification of key MRRDEGs **(A)**. The feature gene importance for the XGB model. **(B)** Statistical graph of variable contribution in SHAP analysis. **(C)** Boxplots showed the residuals of GLM model. **(D)** Cumulative residual distribution of GLM model. **(E)** ROC analysis of GLM and XGB model based on five-fold cross-validation in the testing cohort. **(F)** The important features in GLM models. DN, Diabetic nephropathy. XGB, eXtreme Gradient Boosting. GLM, Generalized Linear Model.

In this study, we utilized four different machine learning algorithms: RF, SVM, XGBoost, and GLM. The RF algorithm was utilized to develop the model, leading to the identification of 40 genes (Figures 4A,B). Subsequently, the SVM algorithm was applied to select the 10 genes with the highest accuracy (AUC = 0.949) (Figures 4C–F). Furthermore, the XGBoost algorithm was used to construct the model, resulting in the selection of 10 genes (Figures 5A,B). Finally, the GLM algorithm was used to screen the key genes, and six genes were identified as *UEDC2*, *CXCR2*, *ATF3*, *GDF15*, *CEBPD* and *NAMPT* (Figures 5C–F).

### 3.5 Diagnostic model construction and evaluation

**FIGURE 6:**
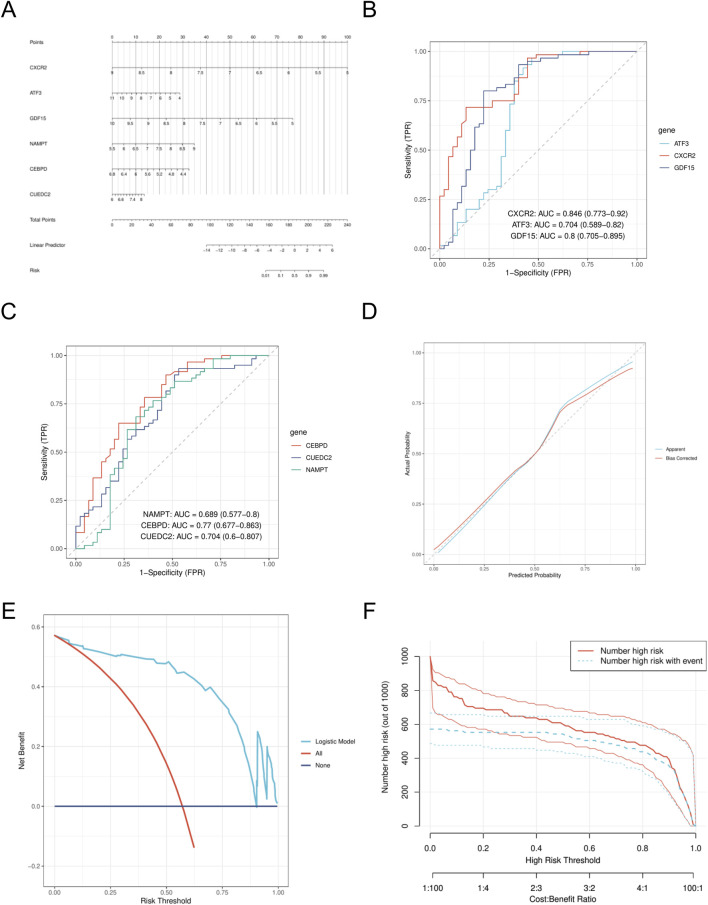
Construction of nomogram model **(A)**. Nomogram. **(B)** ROC analysis of *CXCR2*, *ATF3*, and *GDF15*. **(C)** ROC analysis of *CEBPD*, *CUEDC2* and *NAMPT*. **(D)** calibration curve. **(E)** DCA curves. **(F)** CIC curve. DCA, decision curve analysis; CIC, clinical impact curve.

We created a nomogram model (Figure 6A) and assessed the diagnostic performance of genes using a six-gene ROC analysis. *CXCR2* showed the highest AUC of 0.846 (Figure 6B), while the other genes had AUCs of 0.704, 0.8, 0.689, 0.77, and 0.704 (Figures 6B,C). Calibration curve analysis confirmed that predicted rates matched observed rates well (Figure 6D). Decision curve analysis (DCA) indicated that the logistic regression model with the six diagnostic factors was stable at high-risk thresholds (Figure 6E). In the high-risk threshold range of 0.4–1, clinical impact curves showed strong predictive ability, as the “number of people at high risk” closely matched the “number of people at high risk of having an event” (Figure 6F).

### 3.6 Immune infiltration analysis

**FIGURE 7:**
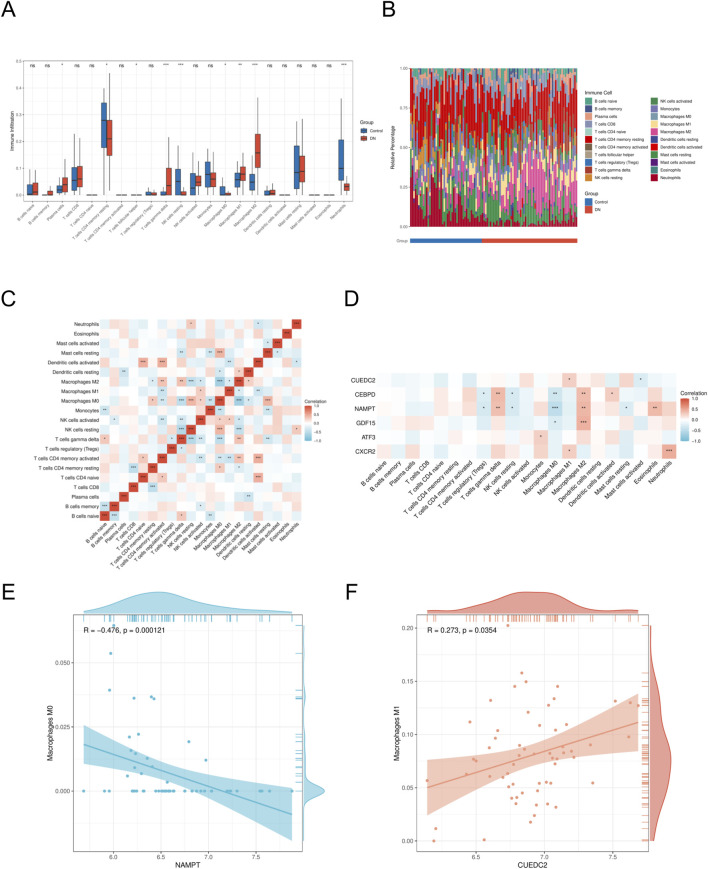
Immune cell infiltration analyses **(A)**. Immune cell distribution map in DN. **(B)** boxplot showing the comparison of 22 kinds of immune cells between DN and the control group. **(C)** heatmap representing the associations of the differentially infiltrated immune cells with immune cells. **(D)** heatmap representing the associations of the differentially infiltrated immune cells with the six hub genes. **(E)** Scatter plot of correlation between *NAMPT* and immune cell. **(F)** Scatter plot of correlation between *CUEDC2* and immune cell. DN, Diabetic nephropathy. The symbol * is equivalent to P < 0.05, which is statistically significant. The symbol ** is equivalent to P < 0.01, which is highly statistically significant. The symbol *** is equivalent to P < 0.001 and highly statistically significant.

To investigate potential changes in the immune system between DN patients and controls, we conducted immune cell characterization utilizing the CIBERSORT algorithm. Our analysis was designed to elucidate variations in the composition of the immune cell population. The findings revealed significant disparities in the proportions of various immune cell types between the DN and control (Figures 7A,B). Notably, the disease group exhibited an elevated presence of M1 macrophages, M2 macrophages, and gamma delta T cells relative to the control group, indicating that alterations in the immune system might be critically involved in the development of DN. Furthermore, interactions among different immune cell types were evident (Figure 7C). The microenvironment of DN was evaluated using the CIBERSORT algorithm, and the relationship between key genes and immune cells was investigated using Spearman’s correlation analysis (p < 0.05, |correlation coefficient|>0.3). Significant associations were identified between most key genes and immune cell components. A notable observation was the positive correlation identified between *CUEDC2* and M1 macrophages. Additionally, *CXCR2* demonstrated a significant positive correlation with neutrophils. In contrast, a significant negative correlation was observed between *NAMPT* expression and the M0 macrophage population (Figures 7D–F).

### 3.7 Single-sample gene set enrichment analysis (ssGSEA)

**FIGURE 8:**
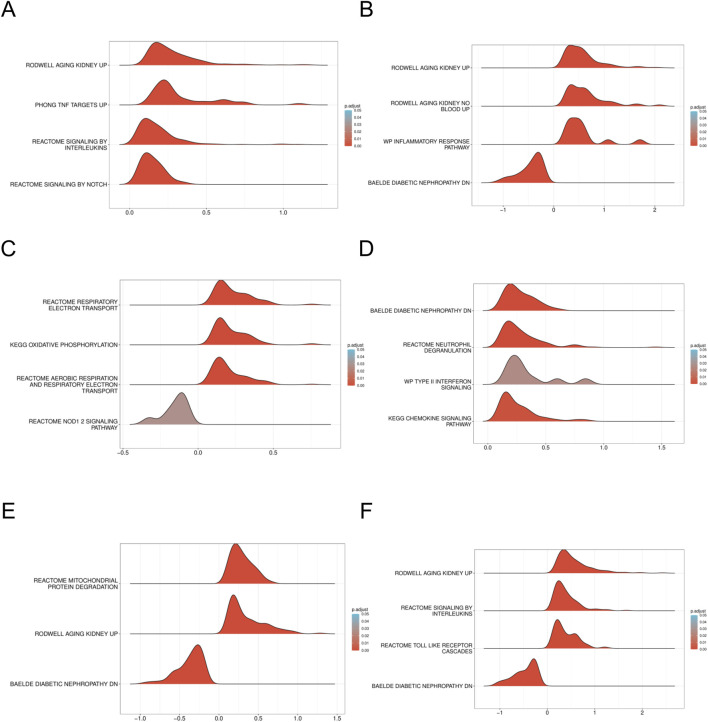
GSEA of DN Datasets dataset **(A)**. GSEA for the *AFT3* in DN. **(B)** GSEA for the *CEBPD* in DN. **(C)** GSEA for the *CUEDC2* in DN. **(D)** GSEA for the *CXCR2* in DN. **(E)** GSEA for the *GDF15* in DN. **(F)** GSEA for the *NAMPT* in DN. GSEA, Gene set enrichment analysis. DN, Diabetic nephropathy. The screening criteria of gene set enrichment analysis (GSEA) were p. Adj< 0.05 and FDR value (q value) < 0.25.

To elucidate the enrichment pathways associated with the characterized genes, we conducted a ssGSEA. Target genes were ranked, and groups with high and low expression were delineated based on expression level differences. The enrichment degree was evaluated by calculating the cumulative score for the target gene set within the ranked list. The GSEA results indicated significant enrichment in pathways such as WP_INFLAMMATORY_RESPONSE_PATHWAY, REACTOME_SIGNALING_BY_INTERLEUKINS, and cellular metabolism-related pathways including KEGG_OXIDATIVE_PHOSPHORYLATION, along with other biologically relevant functions and signaling pathways (Enrichment score >0.5, p < 0.001) (Figures 8A–F). Normalized enrichment scores (NES), false discovery rates (FDR qvalues), and P-values for all pathways are included in Supplementary Table S1, S4.

### 3.8 ceRNA networks and expression verification

**FIGURE 9:**
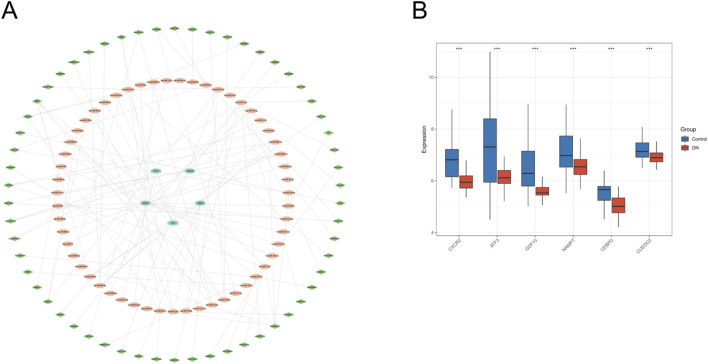
ceRNA network and expression verification. **(A)** ceRNA network of key genes. **(B)** Expresion level of key genes in DN_datasets. ceRNA, competing endogenous RNA. DN, Diabetic nephropathy. The symbol * is equivalent to P < 0.05, which is statistically significant. The symbol ** is equivalent to P < 0.01, which is highly statistically significant. The symbol *** is equivalent to P < 0.001 and highly statistically significant.

The miRNA-mRNA interactions were obtained from TargetScan and subsequently screened to identify miRNAs interacting with five specific genes, resulting in 36 overlapping targeting relationships. Additionally, the StarBase database was employed to predict 82 miRNA-targeted lncRNAs. The datasets were subsequently integrated into Cytoscape software to generate a ceRNA regulatory network (Figure 9A). This network offers insights into the potential regulatory mechanisms driving metabolic reprogramming in DN. We analyzed the expression differences of six key genes in the DN_Datasets dataset between the DN and control groups using the WilCoxon rank-sum test, and the results of the expression difference analysis were presented by the subgroup comparison graph (Figure 9B). The results showed that in the DN_Datasets dataset, the expression of the six key genes was statistically significantly different between the DN and Control groups (P < 0.05). Fold-change and effect size are included in Supplementary Table S5.

### 3.9 qRT-PCR

**FIGURE 10:**
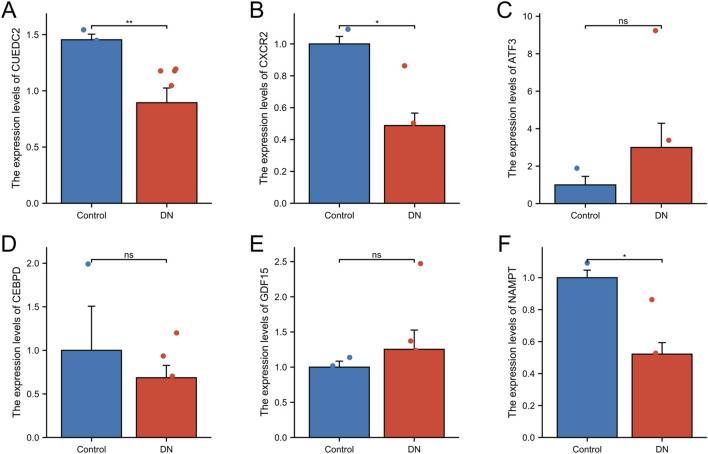
qRT-qPCR. **(A-F)** RT-qPCR validation of relative expression of key genes. The symbol * is equivalent to P < 0.05, which is statistically significant. The symbol ** is equivalent to P < 0.01, which is highly statistically significant. The symbol *** is equivalent to P < 0.001 and highly statistically significant.

The expression levels of the key genes in DNA and control blood samples were examined using qRT-PCR. The findings indicated significant differences in the expression levels of three genes (*CUEDC2*, *CXCR2*, and *NAMPT*), and the differences were statistically significant (P < 0.05). Specifically, the expression of *CUEDC2*, *CXCR2*, and *NAMPT* exhibited lower expression in the DN group relative to the control group. In contrast, no significant difference in expression was observed for *ATF3*, *GDF15*, or *CEBPD* between the DN and control groups (Figures 10A–F).

## 4 Discussion

In the context of DN, renal cells experience substantial metabolic reprogramming, characterized by a transition from mitochondrial oxidative phosphorylation to glycolysis. This metabolic shift is believed to be instrumental in the development and progression of DN (Wang et al., 2022b). The rising prevalence of DN necessitates an urgent investigation into its underlying mechanisms and potential therapeutic targets. In this study, we aimed to clarify the crucial function of metabolic reprogramming in the development of DN using a bioinformatics analysis. We identified 256 MRRDEGs, and GO analysis indicated that these genes were significantly enriched in biological processes related to insulin signaling, carbohydrate catabolism, and glucose control. These observations are consistent with previous studies demonstrating a metabolic disorder in the pathogenesis of DN (Khoshjou and Dadras, 2014). KEGG pathway analysis showed that these genes are mainly linked to the MAPK signaling pathway, which is crucial in DN progression. Hyperglycemia triggers MAPK cascades (ERK/JNK/p38), leading to renal inflammation, fibrosis, apoptosis, and oxidative stress (Thongrung et al., 2025). This pathway connects metabolic signals to tissue damage, making it a key mechanism in DN, aligning with prior research (Wang et al., 2021; Han et al., 2020). We combined WGCNA, Cytohubba (Degree/MCC), and four machine learning algorithms (XGBoost, SVM-RFE, LASSO, Random Forest) to overcome individual model limitations. WGCNA finds co-expression modules but needs hub gene refinement, Cytohubba identifies network hubs but relies on PPI completeness, and machine learning algorithms provide strong feature selection but have biases. We prioritized genes identified by at least two Cytohubba methods and 3 ML algorithms to ensure robust biomarker selection. This approach led to the identification of six key genes: *CXCR2*, *ATF3*, *GDF15*, *NAMPT*, *CEBPD*, and *CUEDC2*. To ascertain the diagnostic utility of the core genes, we assessed their diagnostic performance by constructing the nomogram. Further investigation involved examining immune infiltration in DN using the CIBERSORT algorithm, and evaluating the correlation between key genes and infiltrating immune cells to reveal relevant immune mechanisms. Additionally, we predicted the ceRNA regulatory network to further clarify the regulation of these core genes. While bioinformatics models provide valuable insights, experimental validation is essential for confirming the therapeutic potential of the identified biomarkers. Lastly, qRT-PCR was performed to confirm that *CXCR2*, *NAMPT*, and *CUEDC2* could potentially serve as biomarkers for the clinical diagnosis and risk evaluation of DN patients. *CXCR2* and *CUEDC2* predominantly regulate inflammatory pathways, including NF-κB signaling and macrophage polarization, whereas *NAMPT* plays a critical role in linking metabolic dysfunction (NAD + depletion and mitochondrial impairment) to inflammatory responses and oxidative stress, crucial for DN progression.

*CXCR2*, a receptor that mediates the effects of specific chemokines, functions as a signaling molecule and exerts a key influence on inflammation and tissue damage (Leslie et al., 2022). The deletion of *CXCR2* has been demonstrated to significantly enhance renal function in mice with DN, while concurrently inhibiting the activation of the NF-κB signaling pathway. This pathway is known to regulate inflammation, restore the endothelial glycocalyx, and mitigate DN, as evidenced by a study utilizing a mouse model with a specific knockout of the *CXCR2* gene (Cui et al., 2024). Furthermore, another study suggests that the IL-8-CXCR1/2 axis may contribute to DN by inducing podocyte injury (Loretelli et al., 2021). Inhibition of CXCR1/2 resulted in reduced proteinuria, decreased thylakoid dilatation, and diminished podocyte apoptosis and DNA damage in diabetic mice. Given the observed significant downregulation of *CXCR2* in the DN group, it is suggested that *CXCR2* may not only be involved in the progression of the disease but may also represent the body’s attempt to ameliorate the condition by reducing the activity of the *CXCR2* signaling axis. This complex association underscores the multifaceted role of CXCR2 in maintaining kidney health. Nicotinamide phosphoribosyltransferase (*NAMPT*), an essential enzyme, is pivotal in the biosynthetic pathway of nicotinamide adenine dinucleotide (NAD+), a molecule integral to renal physiology and central to cellular energy metabolism and redox balance. Research indicates that the downregulation of *NAMPT* results in reduced NAD + levels, while *NAMPT* deficiency leads to an overload of mitochondrial ribosomes. This overload impairs the translation of proteins associated with the mitochondrial inner membrane’s oxidative phosphorylation complexes I (CI), III (CIII), IV (CIV), and V (CV), culminating in mitochondrial dysfunction. Furthermore, *NAMPT* downregulation is linked to diminished expression of the transcriptional repressor HIC1, which exacerbates mitochondrial ribosome overload and contributes to diabetic albuminuria and type IV collagen deposition (Hasegawa et al., 2024). These findings imply a protective role of *NAMPT* in DN, aligning with our study’s conclusions. *CUEDC2* is a protein characterized by the presence of the CUE structural domain, which is integral to various biological processes, including the cell cycle, inflammation, and tumorigenesis. Nonetheless, the involvement of *CUEDC2* in the onset and progression of DN remains to be elucidated. Previous studies have indicated that *CUEDC2* may influence bone formation and regeneration through the regulation of the SOCS3-STAT3 pathway (Kim et al., 2020). Additionally, *CUEDC2* has been associated with the oxidative capacity of cardiomyocytes, impacting their oxidative stress response by modulating the stability of GPX1 (Jian et al., 2016). In the context of DN, oxidative stress and inflammation are critical components of its pathophysiological process. Therefore, *CUEDC2* may contribute to the pathogenesis of DN by modulating oxidative stress and the inflammatory response.

The polarization state of macrophages, specifically the equilibrium between M1 pro-inflammatory macrophages and M2 anti-inflammatory macrophages, plays a crucial role in the pathogenesis of DN. Research indicates that excessive activation of M1 macrophages intensifies renal inflammation and fibrosis, while M2 macrophages aid in mitigating these pathological alterations (Yuan et al., 2020; Xie et al., 2020). Our immunological analyses further elucidated distinct patterns of immune infiltration within DN tissues, characterized by an increased presence of M1 macrophages, M2 macrophages, and T cells gamma delta. This altered immune landscape indicates a dynamic interplay of immune responses within DN, particularly marked by the heightened infiltration of T cells gamma delta. T cells gamma delta represent a unique subset of T cell populations with specialized functions, playing a crucial role in immunosurveillance and tissue repair (Ribot et al., 2021). Previous studies have demonstrated that T cells gamma delta can modulate the activity of other immune cells through cytokine secretion and direct cell-cell interactions (Ziegler, 2004). In DN tissues, increased T cells gamma delta likely regulate local immune responses and aid tissue repair. *CUEDC2* positively correlates with M1 macrophages, indicating a role in macrophage polarization and inflammation. *CXCR2* is significantly positively correlated with neutrophils, which is consistent with previous studies. *CXCR2* can recruit neutrophils (Holloman et al., 2024). *NAMPT* negatively correlates with M0 macrophages, suggesting its role in their regulation. M0 macrophages’ differentiation into M1 or M2 influences disease progression (Chen et al., 2018), and *NAMPT* downregulation may boost M0 activation.

Our GSEA analysis confirms that metabolic reprogramming drives DN progression through coordinated activation of inflammatory and metabolism. The dysregulation of *CXCR2*, *NAMPT*, and *CUEDC2*, as identified and validated in this study, represents key molecular intersections within this pathogenic network. Further study of these genes, including their immune connections and interactions with miRNAs and lnRNAs, could guide future DN targeting and immunotherapy focused on correcting metabolic-immune dysregulation. We plan to investigate their mechanisms in DN using molecular biology experiments.

Our study identified *CUEDC2*, *NAMPT*, and *CXCR2* as potential MRRDEGs crucial in DN development. These genes showed high diagnostic value in ROC analysis, suggesting their potential as biomarkers for early DN diagnosis. Their roles in immune cell infiltration and metabolic reprogramming highlight their diagnostic and therapeutic potential. Targeting these genes may offer new strategies to slow or reverse DN progression. However, the study has limitations (Thipsawat, 2021): The current analysis of specific markers in the plasma of DN patients is still in its early stages. To improve the rigor and generalizability of our results, we intend to increase our sample size in future research. Moreover, we plan to utilize additional validation techniques, including Western blotting and ELISA, to confirm the RT-qPCR data and ensure that the observed alterations in gene expression precisely mirror the actual plasma levels of these markers. These further experiments will yield more substantial evidence to substantiate our findings regarding DN biomarkers (Levin et al., 2020). Bias in dataset selection can arise from sample variability, which future research could mitigate by using more uniform datasets or cross-validation. Even with batch-effect correction methods like ComBat, differences between datasets (such as platforms or cohorts) may still cause bias. Future studies should involve larger prospective cohorts (Thomas et al., 2015). Theoretical ceRNA regulatory networks need experimental validation to confirm their therapeutic potential. Both predicted ceRNA networks and CIBERSORT-inferred immune proportions are computational and require experiments, such as luciferase assays for miRNA interactions and flow cytometry/IHC for immune cells, to verify their biological and therapeutic relevance.

## 5 Conclusion

In conclusion, *CXRC2*, *NAMPT*, and *CUEDC2* were identified as key genes involved in DN metabolic reprogramming through GEO database analysis and confirmed by qRT-PCR. These insights improve our understanding of DN’s molecular mechanisms and support future research into new therapeutic strategies targeting these genes. More experiments and integrated multi-omics data are required to confirm these findings.

## Data Availability

The original contributions presented in the study are included in the article/Supplementary Material, further inquiries can be directed to the corresponding authors.
