# Supplementary material for: Investigating the metabolic reprogramming mechanisms in diabetic nephropathy: a comprehensive analysis using bioinformatics and machine learning
Source: Front Cell Dev Biol. 2025 Aug 29;13:1630708. doi: 10.3389/fcell.2025.1630708 (PMC12426288; doi:10.3389/fcell.2025.1630708)
Supplement: Supplementary file 4 [file Table1.docx]

## Table 1. GEO Dataset Information list

|  | GSE30528 | GSE96804 | GSE30529 |
| --- | --- | --- | --- |
| Platform | GPL571 | GPL17586 | GPL571 |
| Experiment type | Expression profiling by array | Expression profiling by array | Expression profiling by array |
| Species | Homo sapiens | Homo sapiens | Homo sapiens |
| Tissue | glomeruli | glomeruli | glomeruli |
| Samples in Control group | Control （13） | Control（20） | Control （10） |
| Samples in Disease group | DN（9） | DN（41） | DN（12） |
| Reference | Transcriptome analysis of human diabetic kidney disease. | Dissection of Glomerular Transcriptional Profile in Patients With Diabetic Nephropathy: SRGAP2a Protects Podocyte Structure and Function. | Transcriptome analysis of human diabetic kidney disease. |

DN, Diabetic nephropathy.
