# Supplementary figures and images for: Investigating the metabolic reprogramming mechanisms in diabetic nephropathy: a comprehensive analysis using bioinformatics and machine learning
Source: Front Cell Dev Biol. 2025 Aug 29;13:1630708. doi: 10.3389/fcell.2025.1630708 (PMC12426288; doi:10.3389/fcell.2025.1630708)

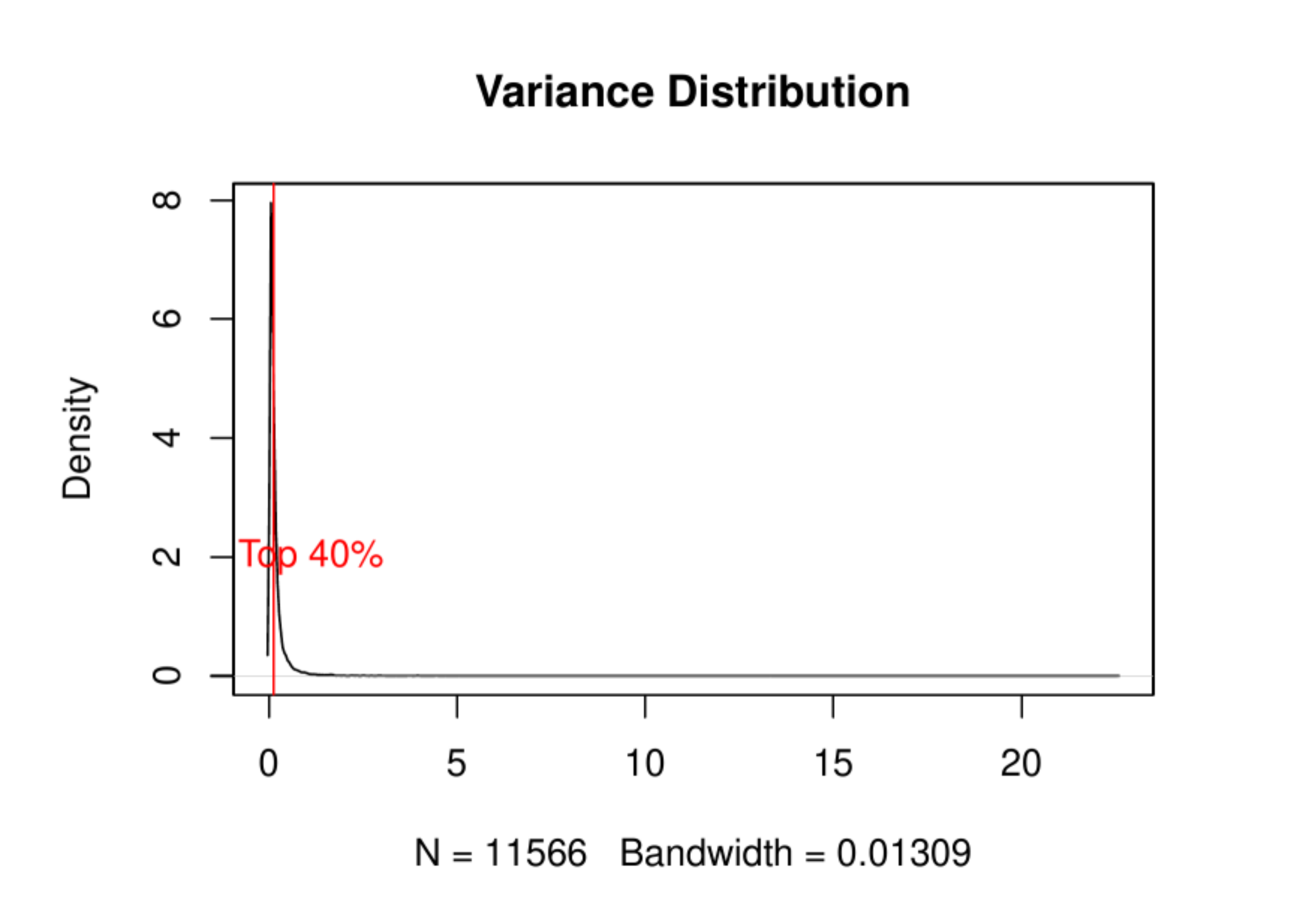

Supplement: Supplementary file 6 [file Image1.tif]
