# Supplementary material for: Investigating the metabolic reprogramming mechanisms in diabetic nephropathy: a comprehensive analysis using bioinformatics and machine learning
Source: Front Cell Dev Biol. 2025 Aug 29;13:1630708. doi: 10.3389/fcell.2025.1630708 (PMC12426288; doi:10.3389/fcell.2025.1630708)
Supplement: Supplementary file 7 [file Table2.docx]

**Table 8.** **Primer sequences for quantitative real-time PCR**

| Gene Names | Forward （5'-3') | Reverse (3ʹ-5ʹ) |
| --- | --- | --- |
| CXRC2 | CTCCCTTTCATAGGTCACAG | AAACTTAAATCCTGACTGGGTC |
| CUEDC2 | GGAACAAAGAGAACCTGCA | CTTCTTTGAGCATTTCGGG |
| NAMPT | GAAATGTTCTCTTCACGGTGG | GACTGAACAAGAATAGTCTCAATCC |
| ATF3 | AGAAGGAGAAGACGGAGTG | TATGCAGGTCTTCAGGACC |
| GDF15 | GCTGGGAAGATTCGAACAC | ACTTCTGGCGTGAGTATCC |
| CEBPD | AGAAGTTGGTGGAGCTGTC | GCAGCTGCTTGAAGAACTG |
